# Human Prion Disease: Pathogenesis, Diagnosis and Public Health

**DOI:** 10.3390/v18020216

**Published:** 2026-02-06

**Authors:** Paola Bellini, Francesco Ruggiero, Andrea Benedetti, Carlo W. Cereda, Claudio Gobbi, Giovanni Bianco, Marco Bongiovanni

**Affiliations:** 1 Division of Infectious Diseases, Ente Ospedaliero Cantonale, 6900 Lugano, Switzerland; paola.bellini01@gmail.com; 2 Neurology Clinic, Neurocenter of Southern Switzerland, Ente Ospedaliero Cantonale, 6903 Lugano, Switzerland; francesco.ruggiero@eoc.ch (F.R.); carlo.cereda@eoc.ch (C.W.C.); claudio.gobbi@eoc.ch (C.G.); giovanni.bianco@eoc.ch (G.B.); 3 Department of Diagnostic and Interventional Neuroradiology, Neurocenter of Southern Switzerland, Ente Ospedaliero Cantonale, 6903 Lugano, Switzerland; andrea.benedetti@eoc.ch; 4 Faculty of Biomedical Sciences, Università della Svizzera Italiana (USI), 6962 Lugano, Switzerland

**Keywords:** prion disease, Creutzfeldt–Jakob disease, iatrogenic transmission, RT-QuIC, corneal transplant, public health, rapidly progressive dementia, neurodegeneration

## Abstract

**Background**: Prion diseases represent a group of rare, progressive, and invariably fatal neurodegenerative disorders. Their hallmark is the infectious nature of the misfolded prion protein (PrP^Sc), which propagates by inducing conformational changes in the physiological form (PrP^C). Despite advances in basic science, these disorders still pose major clinical and therapeutic challenges. **Methods**: A narrative review of the scientific literature was conducted across major biomedical databases, including PubMed, Scopus, Web of Science, and Google Scholar, covering publications up to January 2025. In addition, we describe an illustrative clinical case of a young patient with probable iatrogenic Creutzfeldt–Jakob disease following corneal transplantation, used to highlight diagnostic uncertainty and infection-control implications. **Findings**: Evidence confirms that PrP^Sc drives neurodegenerative processes and transmissibility, with phenotypic and genetic variants influencing clinical course and prognosis. From a diagnostic perspective, neuroimaging techniques and cerebrospinal fluid biomarkers have undergone substantial refinement, with RT-QuIC emerging as a highly specific and sensitive assay. Therapeutic options remain unsatisfactory: no treatment has shown a significant impact on survival. However, innovative strategies (including monoclonal antibodies, gene-based interventions, and modulation of PrP^C) represent promising avenues of investigation. **Conclusions**: Prion diseases remain an unresolved challenge at the intersection of neurology and infectious diseases. Earlier diagnosis through advanced biomarkers and continued development of targeted therapies are essential to improve patient management, while the persistence of iatrogenic cases underscores the ongoing relevance of surveillance and preventive strategies in clinical practice.

## 1. Introduction and Epidemiology

Prion diseases, or transmissible spongiform encephalopathies (TSEs), are rare, invariably fatal neurodegenerative disorders caused by the misfolding of the host-encoded cellular prion protein (PrP^C) into its pathological isoform (PrP^Sc) [1]. This conformational change triggers a self-propagating cascade of protein misfolding, aggregation, and accumulation in the central nervous system, leading to spongiform degeneration, astrogliosis, and neuronal loss [2].

Human prion diseases are classified into three main categories: sporadic forms, which constitute most cases; genetic forms, linked to pathogenic *PRNP* mutations; and acquired forms, including variant and iatrogenic Creutzfeldt–Jakob disease (CJD), resulting from exposure to contaminated biological materials or medical procedures [3,4,5,6]. Sporadic CJD (sCJD) accounts for ~85% of cases, with a stable global incidence of 1–2 per million per year [6,7,8,9,10]. Genetic forms are less common and highly heterogeneous, while acquired forms are now exceedingly rare due to strengthened preventive measures [11,12,13,14].

Despite their low prevalence, prion diseases remain clinically and public health–relevant. Long incubation periods, absence of disease-modifying therapies, and potential iatrogenic transmission—particularly via tissue transplantation or inadequately decontaminated surgical instruments—pose ongoing challenges [12,15,16]. Incidence increases with age, with sporadic forms typically manifesting after the sixth decade, although younger patients may be affected in genetic or acquired cases [17,18,19,20,21].

Clinically, prion diseases progress rapidly and exhibit marked phenotypic heterogeneity, including cognitive decline, cerebellar ataxia, movement disorders, psychiatric symptoms, and visual disturbances [22]. Early recognition is critical, yet diagnosis is often challenging due to overlap with other rapidly progressive dementias [23,24]. Recent advances, particularly real-time quaking-induced conversion (RT-QuIC), have improved diagnostic accuracy, enabling highly specific in vivo detection of prion seeding activity [25]. Prognosis remains poor, with progression typically measured in months rather than years [6,26,27].

We conducted a narrative review of the literature up to January 2026 using major biomedical databases (PubMed, Scopus, Web of Science, Google Scholar) and present an illustrative case of a 33-year-old woman with probable iatrogenic CJD following corneal transplantation, highlighting diagnostic and infection-control challenges.

Prion diseases occupy a unique position at the intersection of neurodegeneration and infection, driven by a self-propagating misfolded protein rather than conventional pathogens. Diagnostic advances, particularly RT-QuIC, have reshaped clinical practice and surveillance, yet these rare disorders continue to pose public health challenges due to iatrogenic transmission and long incubation periods associated with medical procedures. This review synthesizes current knowledge on human prion diseases, integrating molecular mechanisms, diagnostics, and public health implications.

## 2. Classification of Prion Diseases (Table 1)

### 2.1. Sporadic Creutzfeldt–Jakob Disease (sCJD)

sCJD is the most common prion disease, typically presenting in the sixth to seventh decade of life [28,29]. Initial symptoms often include rapidly progressive cognitive decline, sometimes accompanied by visual disturbances, gait ataxia, or subtle behavioural changes [28,30]. Myoclonus is frequently observed early, often triggered by startle. Neurological deterioration advances rapidly, with pyramidal and extrapyramidal features, cortical blindness, mutism, and akinetic state [13,28,30,31,32]. Median survival is 4–6 months, making sCJD one of the most aggressive neurodegenerative disorders [28].

Clinical variability correlates with molecular subtype (Table 2) [9,33,34,35] are:

- MM1/MV1: classical, rapid dementia, myoclonus.
- VV2/MV2: early cerebellar signs, slower progression.
- MM2C: predominantly cognitive, “Alzheimer-like” onset.
- MM2T/FFI: insomnia and autonomic failure.
- VV1: slow course, psychiatric or atypical onset.

**Table 1:** Clinical Features and Disease Course of Prion Diseases. Overview of the main clinical manifestations, age of onset, disease duration, and clinical progression across the principal forms of human prion diseases, including sporadic, variant, genetic, and familial phenotypes. The table highlights distinctive patterns such as rapid cognitive decline in sporadic CJD, early psychiatric presentation in variant CJD, and the slower progression typical of genetic and inherited subtypes (FFI, GSS).

Prion Disease Type	Main Clinical Symptoms	Age of Onset	Disease Duration	Clinical Course
sCJD	Rapid cognitive decline, myoclonus, ataxia	50–75 years	4–6 months	Rapid, progressive decline; myoclonus, visual disturbances
vCJD	Psychiatric symptoms (depression, hallucinations), sensory disturbances, ataxia	20–30 years	13–14 months	Slowly progressive, with early psychiatric symptoms
iCJD	Rapidly progressive cognitive decline, Cerebellar Signs, Myoclonus, Visual symptoms, psychiatric symptoms	30–60 years	5–18 months	Rapid cognitive decline, visual symptoms, myoclonus
gCJD	Ataxia, cognitive decline, dementia	30–60 years	Years	Long course, with early onset and genetic mutations (e.g., E200K, P102L)
FFI	Insomnia, autonomic dysfunction, cognitive decline	30–60 years	1–2 years	Sleep disturbances, progressive autonomic failure, thalamic damage
GSS	Ataxia, dementia, amyloid plaques	30–60 years	5–10 years	Slowly progressive, cerebellar ataxia, long disease course

**Table 2:** Summary of the principal molecular subtypes of human prion diseases, with relive clinical phenotypes and neuropathological characteristics. PrPSc = misfolded pathological prion protein; 129M/V = methionine/valine polymorphism at codon 129 of *PRNP* gene; MM, MV, VV = methionine/methionine, methionine/valine, valine/valine at codon 129; Type 1/Type 2 = electrophoretic types of protease-resistant PrPSc; CJD = Creutzfeldt–Jakob disease; kuru plaques = amyloid plaques typical of MV2K subtype. PSWC = Periodic Sharp Wawe Complexes; FFI = fatal familial insomnia.

Molecular Subtype	Codon 129 Genotype	PrPSc Type	Typical Clinical Phenotype	Key Neuropathology	Notes
MM1	Met/Met	1	Rapid dementia, early myoclonus, classic PSWC on EEG	Fine spongiosis, cortical + basal ganglia involvement	Most common subtype
MM2-cortical (MM2C)	Met/Met	2	Slowly progressive dementia, cortical symptoms	Prominent cortical spongiosis	Often MRI cortical ribboning
MM2-thalamic (MM2T/sporadic FFI-like)	Met/Met	2	Sleep disturbances, dysautonomia	Thalamic neurodegeneration	Phenotype resembles FFI
MV1	Met/Val	1	Classic sCJD phenotype	Mixed spongiosis	Intermediate frequency
MV2 (MV2K, MV2C)	Met/Val	2	Ataxia, cerebellar signs, longer course	Kuru plaques (MV2K) or cortical spongiosis (MV2C)	Frequently misdiagnosed initially
VV1	Val/Val	1	Early psychiatric prodrome, slower progression	Cortical involvement	Very rare subtype
VV2	Val/Val	2	Cerebellar variant: prominent ataxia	Spongiform change in cerebellum and basal ganglia	~15% of sCJD cases

### 2.2. Genetic Prion Diseases

Genetic prion diseases are linked to pathogenic *PRNP* mutations and can present with variable clinical pictures, often more heterogeneous and whose duration is more prolonged than sCJD [30,36,37,38,39]. Pathogenic variants in *PRNP* are associated with distinct phenotypes (Table 3):

- E200K and V210I: “classical” *genetic CJD* (*gCJD*), clinically similar to sporadic forms with rapidly progressive dementia and myoclonus [39].
- V180I: slower progression, often mimicking Alzheimer’s disease due to predominant cortical symptoms [30].
- P102L, A117V, and related mutations: *Gerstmann–Sträussler–Scheinker syndrome* (*GSS*), typically with early cerebellar ataxia and long disease course [36].
- D178N coupled with methionine at codon 129: *Fatal Familial Insomnia* (*FFI*), characterized by intractable insomnia, autonomic dysfunction, and thalamic degeneration [37,39].

### 2.3. Iatrogenic Creutzfeldt–Jakob Disease (iCJD)

Iatrogenic CJD is a rare acquired form of prion disease, resulting from medical exposure to contaminated human-derived materials or instruments [12], accounting for less than 1% of all human prion diseases [14]. Most exposures occurred primarily between the 1960s and 1980s, before the adoption of strict prion inactivation standards and modern sterilization procedures [13]. Documented sources of transmission include: human dura mater grafts and other cadaveric preparations [7], contaminated neurosurgical instruments [12], corneal transplants [40] and cadaveric pituitary-derived human growth hormone (hGH) [18]. The incubation period could be extremely long, up to more than 30 years, depending on the route and site of inoculation [5], therefore new iCJD cases may emerge after decades from the exposure.

### 2.4. Variant CJD (vCJD)

vCJD, associated with bovine spongiform encephalopathy (BSE) exposure, presents peculiar characteristics [5]. It typically affects younger individuals (median age 28 years) and presents with psychiatric and sensory symptoms, such as depression, anxiety, social withdrawal, or painful dysesthesias [41]. Neurological decline follows, with ataxia, cognitive impairment, and involuntary movements. The mean survival in usually longer that sCJD, typically from 12 to 24 months [33,34]. Transmission of variant CJD (vCJD) through blood transfusion has been documented, although the overall risk for recipients remains extremely low. The likelihood increases when blood products have not undergone leukodepletion. Continuous epidemiological surveillance and careful assessment of transfusion-related risk remain essential to ensure public health safety [42].

### 2.5. Kuru (Historical)

Kuru, historically described among the Fore people of Papua New Guinea, resulted from ritual cannibalism and provided pivotal evidence of human-to-human transmission of prion diseases. The disease began with progressive cerebellar ataxia, tremors, and dysarthria, often accompanied by marked emotional lability, known as “kuru laughter”. Progression was inexorable, leading to severe motor impairment and death within 6–12 months [43,44]. Although now extinct, Kuru remains central for understanding prion biology and transmission.

## 3. Pathogenesis

The molecular hallmark of prion diseases is the conformational conversion of the cellular prion protein (PrP^C), a host-encoded glycoprotein, into its misfolded isoform (PrP^Sc) [36]. Whereas PrP^C is soluble, protease-sensitive, and normally involved in neuroprotection and synaptic signalling, PrP^Sc is rich in β-sheet structure, protease-resistant, and prone to aggregation [33]. This pathogenic isoform acts as a template, inducing further misfolding of PrP^C in a self-propagating manner [34,35]. The accumulation of PrP^Sc results in neuronal dysfunction, spongiform degeneration, and gliosis, which constitute the neuropathological hallmarks of prion diseases [21,28].

Host genetic factors critically influence disease susceptibility and phenotype. In particular, the polymorphism at codon 129 of *PRNP* (methionine/valine) strongly modulates risk and clinical presentation: nearly all patients with variant CJD are methionine homozygotes, whereas heterozygotes may exhibit atypical or delayed disease manifestations [45,46,47]. The unique pathogenesis of prion diseases—protein misfolding and propagation in the absence of nucleic acids—distinguishes them from other infectious or neurodegenerative disorders and underlies their dual identity as both infectious and protein misfolding diseases [48].

## 4. Main Histopathological Findings

Prion diseases exhibit a characteristic histopathological profile across different forms [9,28]. The most frequently observed abnormalities include spongiosis (vacuolization of the neuropil, variably distributed across cortical and subcortical regions, and representing the most consistent microscopic hallmark) [49]; neuronal loss (diffuse and progressive, correlating with PrP^Sc accumulation) [50]; reactive astrogliosis (astrocytic hyperplasia and hypertrophy, prominent within spongiform areas and quantifiable via Glial Fibrillary Acidic Protein (GFAP) immunostaining) [2]; and abnormal prion protein (PrP^Sc) deposits (detectable by anti-PrP immunohistochemistry, with distribution and morphology varying according to molecular subtype) [3].

PrP^Sc deposits may adopt several histological accumulation patterns, including synaptic, perivascular, amyloid plaque-like (commonly seen in hereditary forms such as Gerstmann–Sträussler–Scheinker disease), Kuru-type plaques in the cerebellum, and perineuronal deposition [2,45,49]. These patterns are influenced by the molecular type of PrP^Sc and the *PRNP* codon 129 polymorphism, resulting in distinct histopathological profiles [36]. Histopathological diagnosis requires post-mortem confirmation using standard stains and PrP-specific immunohistochemistry [50]. In hereditary forms, highly distinctive histopathological patterns can be observed among the various isoforms [30].

This variability correlates with disease duration and progression rate: forms dominated by amyloid deposition generally follow a longer clinical course, whereas those with diffuse spongiosis are associated with rapidly progressive dementia [51]. The combination of spongiosis, neuronal loss, astrogliosis, and PrP^Sc deposition remains the essential histopathological criterion for diagnosis, with molecular and genetic variants shaping the morphology and distribution of lesions [1,2,52]. Integration of morphology, immunohistochemistry, and molecular typing forms the foundation of the modern neuropathological classification of prion diseases [49].

## 5. Clinical Pictures

Prion diseases are rapidly progressive neurodegenerative disorders that typically evolve over weeks to months and are uniformly fatal [53]. Despite shared neuropathological hallmarks, clinical presentation is heterogeneous and strongly influenced by disease subtype and host factors, particularly the *PRNP* codon 129 polymorphism [35,51,54]. From a clinical standpoint, the key diagnostic feature is the combination of rapid progression with multifocal neurological involvement, often affecting cognition, cerebellar function, and movement control within a short time frame.

Most patients develop a rapidly progressive dementia, frequently accompanied by cerebellar ataxia, myoclonus, pyramidal or extrapyramidal signs, and visual symptoms [22,28]. Early manifestations may be subtle and misleading, including psychiatric symptoms, sensory complaints, or sleep disturbances, contributing to frequent misdiagnosis during the initial phase [28]. As the disease advances, neurological deficits become widespread, culminating in akinetic mutism and severe disability.

### Major Diagnostic Clinical Patterns

Clinically, prion diseases can be conceptualized according to a limited number of dominant patterns, which may guide early suspicion:

- Myoclonus, often startle-sensitive, is a frequent supportive sign and may correlate with periodic sharp-wave complexes (PSWC) on EEG, particularly in sporadic CJD [9,55,56,57].
- Seizures are less common but may occur in advanced disease or atypical subtypes [58,59].
- *Cortical/cognitive pattern*: rapidly progressive dementia with aphasia, apraxia, executive dysfunction, or visuospatial impairment (e.g., MM1/MV1, MM2C, V180I) [60,61,62].
- *Cerebellar pattern*: early gait imbalance and ataxia with relatively delayed cognitive decline (often in VV2/MV2 and some acquired cases) [9,28].
- *Psychiatric/sensory pattern*: prominent psychiatric symptoms and painful dysesthesias, characteristic of variant CJD [41,63].
- *Sleep–autonomic pattern*: insomnia, dysautonomia, and neuropsychiatric changes, typical of fatal familial insomnia and related thalamic subtypes [37,64].

Overall, while no single clinical sign is pathognomonic, the rapid progression and early multisystem neurological involvement remain the most valuable clinical “red flags”.

## 6. Diagnostic Workup

Diagnosing prion disease requires integrating clinical suspicion with targeted investigations, while systematically excluding alternative causes of rapidly progressive neurological decline, especially treatable mimics [65]. In practice, diagnostic reasoning is pattern-based: the likelihood of prion disease increases when rapid progression coexists with characteristic MRI changes and/or positive prion amplification assays.

### 6.1. Neuroimaging

MRI is a cornerstone of diagnosis, and diffusion-weighted imaging (DWI) provides the highest sensitivity, often detecting abnormalities before EEG changes or conventional CSF biomarkers become informative [66,67,68]. The most typical imaging signature in sporadic CJD is restricted diffusion in the cerebral cortex (“cortical ribboning”) and/or basal ganglia (caudate/putamen) [69,70]. Preferential involvement of the insula, cingulate gyrus, and frontal cortex with relative perirolandic sparing may support the diagnosis in the appropriate clinical context [66]. Thalamic involvement is less typical in sCJD but is a hallmark of variant CJD (pulvinar sign) and can also appear in selected sporadic molecular subtypes [67,71].

Importantly, early MRI may be normal, particularly in atypical or slowly evolving forms, and serial imaging can therefore be decisive—an issue illustrated by our clinical case [72,73]. 

### 6.2. PET/SPECT

Functional imaging may demonstrate cortical/subcortical hypometabolism (FDG-PET) or hypoperfusion (SPECT), but these modalities remain supportive and are generally less specific than MRI DWI and RT-QuIC [74]. They may nonetheless assist in atypical cases with inconclusive MRI.

### 6.3. EEG

EEG remains useful as a supportive test, particularly in sporadic CJD, where PSWC occur in approximately two-thirds of cases, typically during intermediate disease stages [56,58]. However, PSWC are neither universal nor specific and are uncommon in variant, genetic, and many iatrogenic forms [75,76]. Therefore, EEG is best interpreted as part of an integrated diagnostic framework rather than as a primary diagnostic test.

### 6.4. CSF Biomarkers

CSF biomarkers reflecting neuronal injury (14-3-3 protein, total tau) are frequently elevated in prion disease but lack specificity, as they may be abnormal in other rapidly destructive brain disorders [77,78,79,80]. In contrast, RT-QuIC has transformed diagnostic practice by enabling highly specific in vivo detection of prion seeding activity, with specificity around 99% and sensitivity exceeding 90% in second-generation assays [81,82,83]. RT-QuIC is now considered the most informative CSF test for suspected prion disease and can also be applied to olfactory mucosa brushings [84,85,86]. Emerging biomarkers such as NfL and pNfH may provide complementary information on disease burden but remain mainly research tools [74,87,88].

### 6.5. Genetic Testing

*PRNP* sequencing is essential when genetic prion disease is suspected and should be strongly considered in early-onset cases, atypical phenotypes, or patients with suggestive family history [36]. Codon 129 polymorphism provides relevant prognostic and phenotypic information and is also epidemiologically important [89,90].

## 7. Diagnostic Criteria for Creutzfeldt–Jakob Disease

The diagnosis of prion diseases, including Creutzfeldt–Jakob disease (CJD), relies on an integrated assessment of clinical features, biomarkers, and neuroimaging findings, with post-mortem confirmation required in selected cases [60,91]. The diagnostic criteria for CJD, as defined by the Creutzfeldt–Jakob Disease International Surveillance Network, are based on clinical, instrumental, and biochemical parameters and classify cases as definite, probable, or possible [7].

### 7.1. Diagnostic Criteria for Sporadic Creutzfeldt–Jakob Disease (sCJD)

#### 7.1.1. Definite sCJD

- Progressive neuropsychiatric syndrome with neuropathological, immunocytochemical, or biochemical confirmation of pathological prion protein.

#### 7.1.2. Probable sCJD

One of the following conditions:

- Rapidly progressive cognitive impairment plus at least two of the following clinical features: myoclonus, visual or cerebellar disturbance, pyramidal or extrapyramidal signs, akinetic mutism, and a typical EEG showing periodic sharp-wave complexes (PSWC); or
- Rapidly progressive cognitive impairment plus at least two of the above clinical features and a typical MRI pattern; or
- Rapidly progressive cognitive impairment plus at least two of the above clinical features and a positive CSF 14-3-3 protein; or
- Progressive neuropsychiatric syndrome with a positive RT-QuIC assay in CSF or other tissues;
- Exclusion of alternative diagnoses.

#### 7.1.3. Possible sCJD

- Rapidly progressive cognitive impairment plus at least two of the following clinical features: myoclonus, visual or cerebellar disturbance, pyramidal or extrapyramidal signs, akinetic mutism, with disease duration <2 years.

### 7.2. Diagnostic Criteria for Iatrogenic Creutzfeldt–Jakob Disease (iCJD)

The diagnosis of iatrogenic CJD (iCJD) differs from sporadic CJD primarily by the presence of a clearly identifiable iatrogenic exposure, while clinical presentation and laboratory findings largely overlap [15]. Definitive diagnosis requires neuropathological confirmation, whereas a probable diagnosis may be established based on compatible clinical features, supportive biomarkers, and documented exposure history [92].

International diagnostic criteria for iCJD, as defined by the World Health Organization (WHO) and the International Creutzfeldt–Jakob Disease Surveillance Network [7], include:

#### 7.2.1. Definite iCJD

- Mandatory neuropathological confirmation (autopsy or brain biopsy demonstrating pathological prion protein).

#### 7.2.2. Probable iCJD

- A rapidly progressive neurological syndrome compatible with CJD (e.g., dementia, ataxia, myoclonus, pyramidal or extrapyramidal signs);
- Supportive CSF and instrumental findings (positive 14-3-3 protein or RT-QuIC, MRI hyperintensity in the caudate nucleus, putamen, or cerebral cortex, EEG showing PSWC);
- A documented history of iatrogenic exposure (e.g., cadaveric human growth hormone therapy, dura mater grafts, contaminated neurosurgical instruments, corneal transplantation, or blood transfusion from an infected donor).

Although no diagnostic criteria are uniquely specific to iCJD, the presence of a confirmed iatrogenic exposure is the key element for international classification [7].

### 7.3. Diagnostic Criteria for Familial Creutzfeldt–Jakob Disease (fCJD)

The diagnosis of familial Creutzfeldt–Jakob disease (fCJD) requires a progressive neurological syndrome compatible with CJD—such as rapidly progressive dementia, myoclonus, ataxia, pyramidal or extrapyramidal signs, or visual and psychiatric disturbances—together with a positive family history or the identification of a pathogenic *PRNP* mutation [49,92,93,94,95,96,97].

#### 7.3.1. Definite fCJD

- Neuropathological confirmation (spongiform change, neuronal loss, gliosis, and pathological prion protein deposition), or
- Genetic confirmation of a known pathogenic *PRNP* mutation associated with CJD [92].

#### 7.3.2. Probable fCJD

- A clinical syndrome compatible with CJD;
- A family history of CJD or another prion disease;
- Supportive CSF biomarkers (14-3-3 protein, tau, or RT-QuIC) and/or characteristic MRI findings (hyperintensity in the caudate nucleus, putamen, or cerebral cortex), in the absence of genetic or neuropathological confirmation.

#### 7.3.3. Possible fCJD

- A compatible clinical syndrome and positive family history, without typical biomarker or neuroimaging findings.

The diagnostic workup of suspected fCJD should always include *PRNP* gene sequencing, as more than 20 pathogenic mutations have been identified, often associated with marked clinical heterogeneity and the potential absence of typical EEG or CSF abnormalities. Neuropathological examination usually demonstrates spongiform change, neuronal loss, and pathological prion protein deposition, with variability in lesion distribution and the presence of amyloid plaques [7].

## 8. Differential Diagnosis (Table 4)

Because prion diseases may present with heterogeneous, rapidly progressive syndromes, the differential diagnosis is broad and should prioritize exclusion of treatable or reversible mimics (Table 4) [73]. In clinical practice, the process is most effective when guided by diagnostic patterns and “red flags” rather than by exhaustive exclusion. Features arguing against prion disease include prominent systemic symptoms (fever, inflammatory markers), inflammatory CSF (pleocytosis/marked protein elevation), sustained epileptic activity, major metabolic derangements, and especially clinical improvement after immunotherapy or antiseizure treatment [65,69].

**Table 4:** Overview of the main differential diagnoses of prion disease.

Disease Group	Onset E Progression	Key Clinical Features	MRI Features	Csf Findings	Response to Treatment
Prion diseases	Subacute onset with multidimensional cognitive impairment (weeks/months)	Rapidly progressive dementia, myoclonus, cerebellar signs, akinetic mutism	Cortical ribboning and/or basal ganglia hyperintensity on DWI/FLAIR, restricted diffusion	Usually normal cell count; Increase intotal tau; 14-3-3 (non-specific); RT-QuIC positive	Steady progression, lack of response to treatment
Autoimmune encephalitis	Subacute onset (days/weeks)	Subacute neuropsychiatric symptoms, seizures, cognitive decline, and movement abnormalities,	Limbic or multifocal T2/FLAIR hyperintensities, often non-DWI-restricted	Mild pleocytosis, oligoclonal bands, neuronal antibodies	Response to immunotherapy
Infectious encephalitis	Acute or subacute onset (days/weeks)	Fever, altered consciousness, seizures, systemic infection signs	Temporal lobe involvement (HSV), focal or diffuse inflammatory lesions	Pleocytosis, Increase in protein, pathogen-specific PCR positive	Response to antimicrobials
Toxic–metabolic encephalopathy	Subacute onset (weeks)	Rapidly progressive cognitive decline, ataxia, and ocular or behavioral symptoms	Diffuse cortical or basal ganglia changes, often reversible	Usually normal or mildly abnormal	Identifiable metabolic/toxic trigger, reversibility with correction
Seizure-related conditions (NCSE)	Acute onset (hours/days)	Fluctuating cognition, myoclonus, subtle motor phenomena	Cortical signal changes are possible, often transient	Typically normal	Rapid improvement after antiseizure treatment
Vascular and inflammatory vasculopathies	Acute or stepwise deficits (weeks)	Headache, blood pressure fluctuations	Vascular distribution, vasogenic edema, reversibility (PRES/RCVS)	The variable may show inflammation	Response to steroids or immunotherapy
Neoplastic/paraneoplastic disorders	Subacute (weeks/months)	Neuropsychiatric decline, known malignancy	Mass lesions, leptomeningeal enhancement, atypical diffusion	Elevated protein, malignant cells or antibodies	Treatable etiology, oncologic context, inflammatory or malignant CSF
Rapidly progressive neurodegenerative dementias	Subacute onset with rapidly progressive dementia (weeks/months)	Cognitive decline with atypical rapidity	Atrophy or non-specific changes	Neurodegenerative biomarkers; RT-QuIC negative	Slower evolution, lack of response to treatment

### 8.1. Immune-Mediated Encephalopathies

Autoimmune encephalitis represents one of the most relevant and treatable mimics of prion disease [98]. It may present with rapidly progressive cognitive and behavioral changes, seizures, and movement disorders. Clinical clues supporting an autoimmune etiology include prominent psychiatric symptoms, frequent seizures, dyskinesias, autonomic instability, or hyponatremia in the appropriate setting [59,99,100,101]. Supportive investigations include MRI features consistent with encephalitis and inflammatory CSF findings such as mild pleocytosis or oligoclonal bands, with confirmation obtained through serum/CSF antibody testing [65,69,95]. Because early recognition has major therapeutic implications, autoimmune encephalitis should be systematically considered in all cases of rapidly progressive dementia.

### 8.2. Infectious Encephalitis

Infectious encephalitis—particularly HSV encephalitis—must be promptly excluded given its severity and treatability [102,103]. Fever, seizures, altered consciousness, temporal lobe abnormalities on MRI, and CSF pleocytosis strongly suggest an infectious etiology and warrant urgent PCR-based testing [102,103,104,105]. Other infectious mimics include viral, bacterial, fungal, and opportunistic infections, especially in immunocompromised patients [106]. Clinical context, MRI distribution, and CSF profile are essential to differentiate these conditions from prion disease.

### 8.3. Toxic–Metabolic and Seizure-Related Conditions

Toxic–metabolic encephalopathies (hepatic, uremic, electrolyte-related, endocrine, nutritional, and drug/toxin-related) may produce rapidly progressive cognitive decline with ataxia, behavioral symptoms, or myoclonus [107]. Several metabolic conditions can also generate cortical diffusion abnormalities on MRI, creating radiological overlap with prion disease [66]. Therefore, baseline laboratory evaluation—including electrolytes, liver/kidney function, thyroid function, vitamins, and ammonia—remains mandatory in rapidly progressive syndromes [108,109].

Seizure-related conditions, especially non-convulsive status epilepticus, represent another key mimic, with fluctuating cognition and myoclonus. Because peri-ictal MRI diffusion restriction can resemble cortical ribboning, urgent EEG is essential; improvement after antiseizure treatment strongly argues against prion disease [56,58].

### 8.4. Vascular and Inflammatory Vasculopathies

Several vascular disorders may mimic prion disease clinically or radiologically. Hypoxic–ischemic injury, profound hypoglycemia, and selected vasculopathies can produce widespread cortical abnormalities, while deep venous infarcts may involve the thalamus and mimic thalamic-predominant prion syndromes [66]. Reversible cerebral vasoconstriction syndrome (RCVS) and posterior reversible encephalopathy syndrome (PRES) may present with acute/subacute encephalopathy and MRI changes, but typically show a fluctuating course and potential reversibility [110,111,112,113]. Correlation with vascular imaging and clinical context is therefore crucial.

### 8.5. Neoplastic and Paraneoplastic Disorders

Neoplastic disorders remain important mimics because they are potentially treatable. Primary CNS lymphoma and leptomeningeal carcinomatosis may present with subacute cognitive decline and, in some cases, restricted diffusion on MRI [114]. Paraneoplastic neurological syndromes and immune-mediated encephalopathies related to cancer therapy (including immune checkpoint inhibitors or CAR-T) may also present with rapidly progressive encephalopathy [114,115,116,117,118,119]. In these settings, inflammatory CSF, atypical MRI patterns, and treatment responsiveness support an immune-mediated process rather than prion disease.

### 8.6. Rapidly Progressive Neurodegenerative Dementias

Other neurodegenerative disorders (Alzheimer’s disease, dementia with Lewy bodies, frontotemporal lobar degeneration) may occasionally present with accelerated progression and overlap clinically with prion disease [61]. In such cases, CSF biomarkers are particularly helpful. Conventional markers such as 14-3-3 protein and total tau reflect neuronal injury but lack specificity [79]. In contrast, RT-QuIC provides highly specific evidence of prion seeding activity and has become central in the diagnostic algorithm of rapidly progressive dementia [25,115,120].

## 9. Infection-Control and Procedural Precautions When Prion Is in the Differential Diagnosis

Prion diseases pose minimal risk through casual contact or routine patient care; however, they entail a distinct iatrogenic risk when invasive procedures involve tissues with high infectivity, particularly the brain, spinal cord, and posterior eye. Prions are notably resistant to standard sterilization, and incomplete decontamination may result in persistent contamination of re-usable instruments.

When prion disease is a plausible differential diagnosis—even prior to confirmation—early implementation of precautionary measures is recommended [121]. Practical principles include preferential use of single-use instruments when feasible, clear labeling and traceability of potentially contaminated materials, and early notification of pathology, operating theatre, and sterile processing services before procedures involving high-risk tissues. Instruments exposed to high-infectivity tissues should not be reused unless they undergo validated prion decontamination protocols; devices that cannot be reliably decontaminated may require withdrawal from service. These measures align with CDC, WHO, and national guidance emphasizing precaution, instrument stewardship, and surveillance [122].

## 10. Therapeutic Strategies

Currently, no approved therapies can alter the course of prion diseases, and patient management remains purely symptomatic and palliative [9,123,124]. Quinacrine, tested in a randomized controlled trial in sporadic CJD and the prospective PRION-1 cohort study, failed to show sustained clinical benefit, with only a minority of patients exhibiting transient responses. This lack of efficacy has been attributed to poor penetration into the central nervous system and the inherent resistance of pathogenic prion conformations [125,126,127]. Tetracyclines have been investigated preclinically for their ability to interact with pathological PrP, reduce protease resistance, and interfere with amyloid aggregation. While these compounds prolonged survival in experimental models, their effectiveness has not been confirmed in humans; they are therefore not considered therapeutic agents, although they remain of interest for preventive measures, decontamination protocols, and anti-aggregant applications [9,128,129].

A dual-mechanism approach has recently been proposed, aiming to overcome strain-dependent drug resistance by targeting multiple functional regions of PrP simultaneously, representing a promising conceptual framework for future drug development [130]. Experimental strategies under investigation include passive immunotherapy with anti-PrP^C monoclonal antibodies, such as PRN100, which have demonstrated acceptable safety and CNS bioavailability but no proven clinical efficacy to date [131,132,133]. Approaches to reduce PrP^C expression using antisense oligonucleotides (ASOs) or RNA interference are currently in early-phase clinical trials, showing favorable tolerability and minimal neurotoxicity in preclinical studies [133,134]. Additional experimental therapies focus on small molecules that inhibit PrP^Sc aggregation, multi-target compounds, β-sheet breakers, and modulators of autophagy, although none have yet shown clinical benefit. Gene therapy approaches, including CRISPR/Cas9-based strategies, as well as stem cell interventions, remain at the preclinical stage [133,135,136,137,138,139].

Studies in asymptomatic carriers of pathogenic *PRNP* mutations indicate that CSF PrP^C levels remain stable over time and may serve as pharmacodynamic biomarkers, whereas markers of neuronal injury, such as neurofilament light chain and tau, and prion seeding activity measured by RT-QuIC, are less sensitive during the prodromal phase [133]. PRN100 has shown favorable safety and CNS penetration in symptomatic patients, supporting its evaluation in asymptomatic carriers or individuals at very early disease stages [89]. Patient selection for preventive or early-intervention trials relies primarily on the identification of pathogenic *PRNP* mutations, with particular focus on asymptomatic carriers, such as E200K mutation carriers, who may exhibit RT-QuIC positivity one to three years before clinical onset. Phenotypic variability and the absence of robust predictive biomarkers continue to complicate patient stratification and the definition of clinical endpoints [82,136].

In summary, the most promising experimental strategies currently include ASOs and passive anti-PrP^C immunotherapy, both demonstrating encouraging safety profiles, although clinical efficacy remains unproven. Despite advances in RT-QuIC and other protein amplification assays that have markedly improved diagnostic accuracy, significant challenges remain. The development of non-invasive biomarkers capable of predicting disease onset in at-risk individuals, including transplant recipients, and the creation of blood- or skin-based assays to overcome the limitations of CSF testing, are essential for earlier detection and for facilitating preventive therapeutic trials [140]. Future research directions focus on early diagnosis, therapies designed to reduce PrP^C expression, immunotherapy, combination treatment strategies, advanced drug delivery systems, and the integration of artificial intelligence to identify novel therapeutic targets and optimize clinical trial design [9,135,136,141].

## 11. An Emblematic Clinical Case

A 33-year-old woman with an otherwise unremarkable medical history underwent corneal transplantation in 2010 due to complications from a disseminated Pseudomonas aeruginosa infection. She did not require chronic medications thereafter, and there was no family history of neurodegenerative disease or substance abuse.

**Figure 1:**
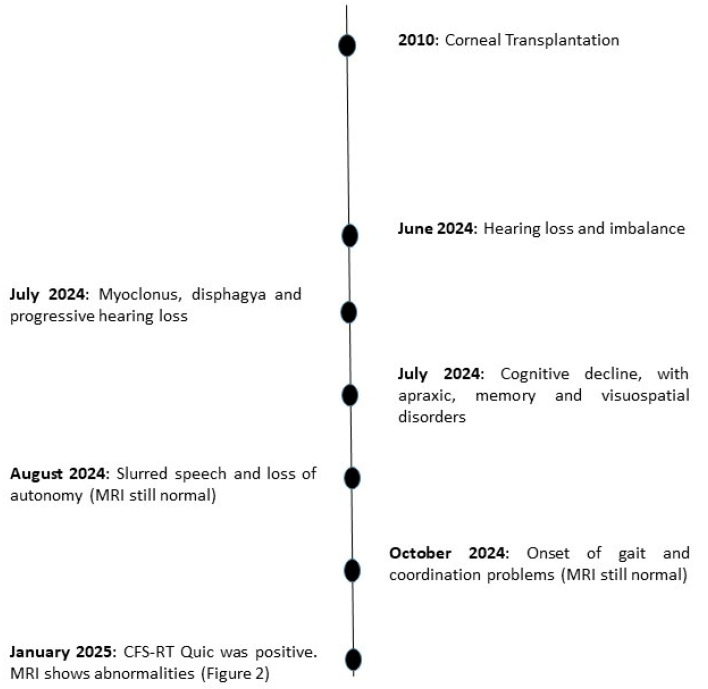
Timeline of symptom onset and diagnostic work-up in a patient with iatrogenic Creutzfeldt–Jakob disease (iCJD).

Her first neurological symptom appeared in early June 2024 as subtle, progressive hearing loss. Within weeks, she developed gait imbalance and unsteadiness, which rapidly evolved into a full cerebellar syndrome with diffuse tremor, dysarthria, gait ataxia, frequent near-falls, and progressive loss of coordination. During the second and third weeks, involuntary myoclonic jerks, predominantly affecting the upper limbs, emerged, alongside dysphagia—particularly for liquids—which interfered with daily activities and contributed to weight loss. Hearing further deteriorated, significantly affecting communication and social interaction. By the fourth week, cognitive and behavioural changes, including psychomotor slowing, forgetfulness, and impaired attention, were noted. Neurological decline accelerated over the next two weeks, leading to increasing dependency in daily activities, slurred speech, and severe gait instability requiring continuous assistance, prompting admission to a neuro-rehabilitation facility. Notably, the patient did not exhibit fever, systemic infectious symptoms, or metabolic disturbances, making a primary systemic, infectious, or metabolic aetiology unlikely (Figure 1).

Initial hospitalization in an Infectious Diseases Department included brain MRI and CSF analysis, as well as extensive infectious screening of serum and CSF. The first MRI in July 2024 was unremarkable, and a second MRI in October 2024 also showed no abnormalities, despite progressive cerebellar and myoclonic symptoms. At that stage, prion disease was considered, but alternative parainfectious causes could not be excluded.

Upon admission to our Neurology Department, further workup included CSF cultures and multiplex PCR testing for neurotropic viruses, as well as bacterial, fungal, and serological testing for syphilis and Lyme disease, all of which were negative. Autoimmune and paraneoplastic panels in serum and CSF, including antibodies for autoimmune encephalitis and classical paraneoplastic syndromes, were also negative. Metabolic and toxicological assessments—including thyroid function, vitamins B1, B12, folate, ammonia, lactate, and comprehensive toxicology—were normal. FDG-PET revealed only an incidental thyroid nodule and no patterns indicative of alternative neurodegenerative, inflammatory, or neoplastic processes, and systemic malignancy was excluded.

**Figure 2:**
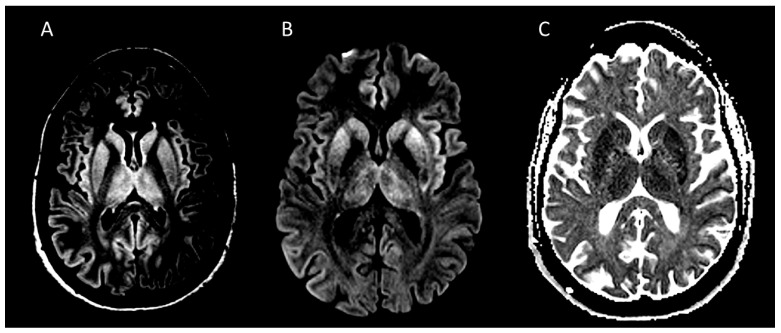
MRI findings in a patient with iatrogenic Creutzfeld-Jacobs Disease. (**A**): Axial FLAIR MRI image showing bilateral hyperintense signals in the basal ganglia, thalami, and cortical insular and cingulate ribbons, with asymmetric involvement of the right temporo-occipital cortex; (**B**): Diffusion-weighted imaging (DWI) demonstrates marked hyperintensity along the cortical ribbon, bilateral putamina, caudate nuclei, and thalami, consistent with restricted diffusion due to prion-induced spongiform change; (**C**): apparent diffusion coefficient (ADC) map reveals signal hypointensity in the same areas, confirming true restricted diffusion and excluding the T2 shine-through effect. The original images are stored at the Institute of Neurology of Southern Switzerland (INSI, Ospedale Civico di Lugano).

A third brain MRI in January 2025, approximately four months after symptom onset, revealed cortical ribboning and cerebellar hyperintensities on DWI and FLAIR sequences, strongly suggestive of prion disease (Figure 2). CSF RT-QuIC testing was positive in all four replicates, confirming prion seeding activity. *PRNP* genetic testing was offered to evaluate for inherited forms but was declined by the family.

Based on the rapidly progressive cerebellar and cognitive syndrome, positive CSF RT-QuIC results, and supportive MRI findings, a probable diagnosis of human prion disease was established. Although the exact etiology could not be confirmed, the patient’s young age and history of corneal transplantation strongly supported iatrogenic Creutzfeldt–Jakob disease.

Management focused on supportive and palliative care, addressing dysphagia, mobility impairment, and myoclonus, with coordinated neuro-rehabilitation and family counselling. The case was reported to the Cantonal Public Health Authority for surveillance. Despite these measures, the patient’s condition progressed relentlessly, consistent with the natural course of prion disease, and she died six months after diagnosis. Autopsy results were not available.

## 12. Lessons Learned from This Clinical Case

Prion diseases represent one of the most challenging entities in neurology and infectious diseases, owing to their rarity, heterogeneous clinical presentations, and the absence of effective disease-modifying therapies. The present case exemplifies how these features translate into tangible diagnostic and public health challenges.

First, it highlights the substantial diagnostic uncertainty that may characterize early-stage prion disease, particularly in young patients with atypical presentations. The initial subacute cerebellar syndrome with myoclonus and dysphagia, combined with repeatedly normal MRI and cerebrospinal fluid findings, favoured alternative diagnoses such as viral or parainfectious encephalitis. Cognitive decline became apparent only at a later stage and may initially have been obscured by the patient’s young age and presumed high cognitive reserve. This case underscores the well-recognized but clinically problematic delay between symptom onset and the emergence of characteristic neuroimaging abnormalities, reinforcing the importance of serial imaging and the integration of highly specific biomarkers such as RT-QuIC, which ultimately proved decisive for diagnosis.

Second, the case illustrates the persistent complexity of etiological attribution even after a diagnosis of prion disease has been established. Although sporadic Creutzfeldt–Jakob disease cannot be excluded, the unusually young age at onset and the early predominance of cerebellar involvement raise the possibility of alternative aetiologies, including genetic or iatrogenic forms. In this context, the history of corneal transplantation is particularly relevant, as incubation periods ranging from one to three decades are well documented in iatrogenic CJD. This observation emphasizes the need for meticulous review of prior medical interventions and, when feasible, the incorporation of *PRNP* genetic testing into the diagnostic workup.

Finally, this case underscores the infection-control and public health implications inherent to diagnostic uncertainty. During the interval in which prion disease is suspected but not yet confirmed, clinicians must balance optimal patient care with precautionary measures related to invasive procedures, specimen handling, and institutional safety protocols. Moreover, the prolonged latency associated with iatrogenic transmission highlights how historical medical practices may continue to generate cases decades later, despite contemporary improvements in safety standards.

Taken together, this case does not merely represent a rare clinical observation but reinforces the central themes of this review: the unique biological nature of prion diseases, the transformative impact of modern diagnostic tools, and the enduring importance of infection-control decision-making and public health surveillance.

## 13. Public Health Implications and Preventive Strategies

Prion diseases remain rare but carry disproportionate public health relevance due to their potential for iatrogenic transmission and the exceptional resistance of prions to standard sterilization procedures [5,12,121,122]. Historically, outbreaks of iatrogenic CJD associated with cadaveric dura mater grafts, pituitary-derived human growth hormone, corneal transplantation, and inadequately decontaminated neurosurgical instruments have driven major advances in surveillance systems and infection-control policies [12,40]. Although the incidence of these forms has markedly decreased following strengthened preventive measures, the risk has not been completely eliminated, largely because incubation periods can extend for decades and because asymptomatic carriers cannot be reliably identified through routine donor screening [5,121].

Current preventive strategies rely on multiple complementary approaches, including strict donor selection criteria, traceability of tissues and instruments, and validated decontamination protocols for high-risk procedures [122,142]. In practice, risk mitigation is particularly relevant for procedures involving tissues with high infectivity, such as brain, spinal cord, and posterior eye structures. When prion disease is suspected, early implementation of precautionary measures—such as preferential use of single-use instruments, quarantine/traceability of exposed devices, and early notification of pathology and sterile processing services—represents a pragmatic approach to prevent inadvertent secondary exposure [121,122,143].

An emerging and increasingly debated aspect concerns the safety of ocular tissue transplantation. Experimental and clinical evidence indicate that prion seeding activity can be detected in ocular tissues, including the cornea, in sporadic CJD, supporting the biological plausibility of transmission through corneal grafting [40]. These findings have prompted discussion on whether preventive strategies should evolve from indirect risk reduction (donor selection and sterilization protocols) toward direct screening of donor tissues using prion amplification assays. In particular, RT-QuIC and PMCA provide high analytical sensitivity and could theoretically be integrated into transplant workflows as a targeted screening tool for higher-risk tissues [144,145]. A notable example is the Czech Republic, where mandatory Western blot testing of brain tissue from corneal donors has been implemented since 2007, with all samples reported negative to date [38]. While broader implementation would require careful evaluation of feasibility, turnaround time, cost, and ethical implications (including false-positive handling), donor screening represents a rational future direction for further reducing residual iatrogenic risk.

Overall, public health management of prion diseases depends on coordinated surveillance, timely reporting, and adherence to infection-control protocols, particularly in settings involving invasive procedures or tissue transplantation. As diagnostic technologies advance, prevention strategies may increasingly shift toward earlier identification of prion seeding activity in donor tissues, complementing existing measures aimed at minimizing iatrogenic transmission [121,122,144].

## 14. Conclusions

Prion diseases remain among the most devastating neurodegenerative disorders, characterized by rapid progression, diagnostic challenges, and a lack of effective therapy. While their overall incidence is low, their unique biologic profile, potential for iatrogenic transmission, and invariably fatal outcome make them a matter of ongoing clinical and public health concern [146].

The present clinical case highlights several critical issues. First, it illustrates the diagnostic complexity inherent to prion disease: early MRI scans may be normal, and diagnosis often relies on advanced biomarkers such as RT-QuIC, combined with serial neuroimaging and exclusion of alternative causes. Second, it raises an etiological dilemma: while the sporadic disease is by far the most common form, the patient’s age, clinical phenotype, and medical history compel consideration of genetic and iatrogenic origins. This uncertainty underscores the importance of integrating genetic testing, thorough anamnesis, and epidemiological context into the diagnostic process [147].

From a public health perspective, the case also reinforces the need to revisit donor screening strategies. Current exclusion criteria based solely on clinical suspicion at the time of death may be insufficient, particularly for tissue grafts with documented transmission risk, such as cornea. As evidence grows regarding the presence of prion seeds in ocular tissues, and as pilot programs in Europe have demonstrated the feasibility of donor tissue testing, it is time to consider whether direct prion screening assays should be incorporated into transplant safety protocols, especially for younger recipients who are at higher risk due to the long incubation periods of prion diseases.

In conclusion, the challenges posed by prion diseases are multifaceted, requiring close collaboration among neurologists, infectious disease specialists, radiologists, and public health authorities to ensure timely diagnosis, optimal patient management, and maximal safety in transplantation and clinical procedures. Continued investment in biomarker development, genetic research, and advanced donor-tissue screening technologies represents the most promising path toward improving patient care and preventing future iatrogenic transmissions.

## Figures and Tables

**Table 3:** Summary of the main genetic subtypes, including sporadic, variant, genetic, and inherited forms. The table reports the associated *PRNP* genotypes, PrP^Sc molecular types, main clinical features, incidence, prognosis, and defining clinic pathological characteristics. Data are derived from recent consensus papers and molecular studies describing prion strain heterogeneity and genotype–phenotype correlations. *PRNP* = prion protein gene; GCJD = genetic Creutzfeldt–Jakob disease; FFI = Fatal Familial Insomnia; GSS = Gerstmann-Sträussler-Scheinker disease; E200K, D178N, P102L, etc. = pathogenic mutations in *PRNP* gene; yrs = years.

Disease/Phenotype	*PRNP* Mutation	Typical Age of Onset	Clinical Features	Notes
Genetic CJD (GCJD)	E200K	50–60 yrs	Rapid dementia, myoclonus	High-penetrance clusters (e.g., Israel, Slovakia)
	V210I	50–70 yrs	sCJD-like, rapid course	Common in Europe
	D178N (with 129V)	40–60 yrs	CJD phenotype	Genotype determines phenotype
	T183A, R208H, R148H	Variable	sCJD-like	Rare mutations
Fatal Familial Insomnia (FFI)	D178N-129M	30–60 yrs	Insomnia, dysautonomia, ataxia	Complete penetrance
Gerstmann-Sträussler-Scheinker (GSS)	P102L	30–60 yrs	Ataxia, dysarthria, long course (years)	Classic GSS mutation
	A117V	30–50 yrs	Early cognitive decline + ataxia	
	F198S	40–60 yrs	Cerebellar + extrapyramidal signs	
				
Other rare *PRNP* mutations	E196A, E196K, Y163X (truncation), Q160X	Variable	Highly heterogeneous	Some cause prion amyloidosis
				
Stop-codon mutations (*PRNP* truncations)	Y145X, Q160X, Y226X, Q227X	20–60 yrs	Amyloidosis, chronic course	Often systemic PrP deposition

## Data Availability

No new data were created or analyzed in this study.

## Abbreviations

PrP	Prion protein
PrPC	Cellular prion protein (normal form)
PrPSc	Scrapie-associated pathological prion protein
TSE	Transmissible spongiform encephalopathy
CJD	Creutzfeldt–Jakob disease
sCJD	Sporadic Creutzfeldt–Jakob disease
gCJD/fCJD	Genetic/familial Creutzfeldt–Jakob disease
vCJD	Variant Creutzfeldt–Jakob disease
iCJD	Iatrogenic Creutzfeldt–Jakob disease
FFI	Fatal Familial Insomnia
GSS	Gerstmann-Sträussler-Scheinker disease
RPD	Rapidly progressive dementia
PRNP	Prion protein gene
129M/V	Methionine/Valine polymorphism at codon 129 of *PRNP*
MM, MV, VV	Methionine/Methionine; Methionine/Valine; Valine/Valine genotypes
Type 1/Type 2 PrPSc	Electrophoretic prion protein types
E200K, D178N, P102L, V180I, etc.	Common *PRNP* pathogenic mutations
PSWCs	Periodic Sharp Wave Complexes (EEG hallmark)
MRI	Magnetic resonance imaging
DWI	Diffusion-weighted imaging
ADC	Apparent diffusion coefficient
FLAIR	Fluid-attenuated inversion recovery
CT	Computed tomography
PET	Positron emission tomography
FDG-PET	Fluorodeoxyglucose positron emission tomography
CSF	Cerebrospinal fluid
RT-QuIC	Real-Time Quaking-Induced Conversion
qRT-QuIC/2G-RT-QuIC	First or second generation RT-QuIC
t-tau	Total tau protein
p-tau	Phosphorylated tau
NfL	Neurofilament light chain
14-3-3	CSF 14-3-3 protein
EEG	Electroencephalography
